# Failure to thrive and severe malnutrition secondary to duodenocolic fistula: A case report

**DOI:** 10.1016/j.ijscr.2022.107259

**Published:** 2022-05-30

**Authors:** Ahmed A. Alburakan, Sulaiman A. Alshammari, Fayez G. Aldarsouni, Thamer A. Nouh

**Affiliations:** a Trauma and Acute Care Surgery Unit, Department of Surgery, College of Medicine, King Saud University, Riyadh, Saudi Arabia; b Department of Surgery, College of Medicine, King Saud University, Riyadh, Saudi Arabia; c College of Medicine, King Saud University, Riyadh, Saudi Arabia

**Keywords:** Duodenocolic fistula, Chronic diarrhea, Abdominal pain, Weight loss, Case report

## Abstract

**Introduction and importance:**

Duodenocolic fistula (DCF) is a rare surgical condition. Patients usually develop chronic nonspecific symptoms. DCF has major consequences on patients' health and quality of life. This report describes an adult male patient with chronic nonspecific gastrointestinal and malnutrition symptoms. After several attempts to establish a diagnosis, DCF was discovered incidentally during abdominal exploration.

**Case presentation:**

We are presenting a case of a 33-year-old male patient with a long-standing history of malnutrition and failure to thrive. He underwent multiple investigations in several hospitals with no definitive diagnosis. All investigations were repeated in our hospital which were inconclusive. Two years after the first presentation to our hospital, the patient presented to the emergency room with bowel obstruction. During his admission, he was thoroughly investigated. His hospital admission included 2 surgeries where eventually a duodenocolic fistula was identified and surgically resected. All the patient's symptoms improved thereafter.

**Conclusion:**

This case highlights some of the obstacles in diagnosing and managing DCF. Early diagnosis is essential to avoid serious complications. Management options should be tailored to the underlying cause and patient conditions. Regardless of the cause of the fistula, surgery is the only treatment.

## Highlights

• Duodenocolic fistula is an infrequent surgical condition.
• Early diagnosis and intervention are the cornerstones of treatment.
• Duodenocolic fistula patients are ill because of malnutrition, malabsorption, and dehydration.
• Regardless of the cause of the fistula, surgery is the only treatment.

## 1 Introduction

Duodenocolic fistula (DCF) is rare; it is defined as the presence of an abnormal tract between the duodenum and the colon [1]. Primary DCF occurs in the absence of abdominal surgery, while secondary DCF occurs after abdominal surgery [2].

Primary DCF is subdivided into benign and malignant. Benign DCF occurs as a consequence of different gastrointestinal diseases, such as peptic ulcer disease, Crohn's disease, and duodenal or colonic diverticula [3]. The manifestations of DCF are vague and non-specific. The most common symptoms are chronic abdominal pain, in approximately 79% of the cases, followed by chronic diarrhea (75%), weight loss (64%), and nausea and vomiting (50%) [4].

The diagnosis of DCF can be challenging because it is so rare and presents with nonspecific symptoms [5]. Upper and lower gastrointestinal imaging with contrast is crucial in its diagnosis [6]. This case report describes an adult male patient who has been suffering from severe malnutrition symptoms for 15 years secondary to DCF. Written informed consent was obtained from the patient for future research and publication. This case has been reported in line with the SCARE 2020 criteria [7].

## 2 Case presentation

A 33-year-old male post office worker with severe malnutrition and failure to thrive for 15 years. He presented to our emergency department (ED) complaining of abdominal pain and persistent vomiting. He was tachycardic on examination. We excluded mechanical bowel obstruction and other surgical causes through clinical assessment and computerized tomography (CT) Abdomen.

**Fig. 1:**
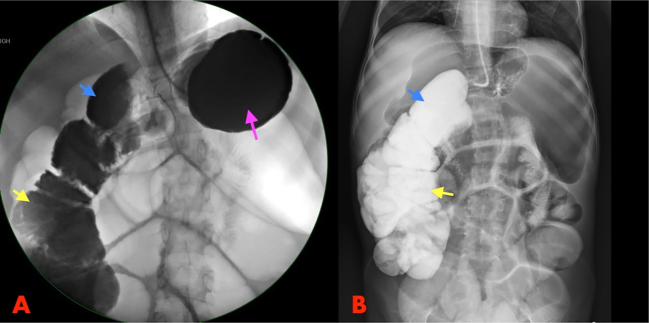
X-ray fluoroscopy of small bowel gastrografin study. (A) The contrast filled the stomach (pink arrow), then directly seen in the hepatic flexure (blue arrow) and ascending colon (yellow arrow). (B) The contrast then moved to the hepatic flexure (blue arrow) and ascending colon (yellow arrow) immediately going from the stomach through the duodenum. The finding is compatible with a fistula between the first part of the duodenum and the hepatic flexure of the colon.

The patient was then managed as a case of non-specific gastritis and referred to outpatient clinic for investigations. A detailed history was obtained from the patient. The patient had undergone bowel resection at age of two years. He had always been suffering from chronic persistent diarrhea (˃5 times/day for 10 years), intermittent abdominal pain, and failure to thrive. In addition, he noticed a significant decrease in his libido. He had no history of jaundice, vomiting, or gastrointestinal bleeding. He had been diagnosed with celiac disease in another hospital based on clinical findings only. He was managed with gluten-free diet for 10 years with no significant improvement. The absence of the typical celiac symptoms mandated re-investigation. Celiac disease was excluded as there was no evidence of the disease on upper and lower endoscopy, histopathology and serology tests. All findings suggested malabsorption syndrome. His height was 146.7 cm and his BMI ranged from 11.6 to 14.7 kg/m^2^. During 2 years follow up in our hospital, he had 14 visits to ED and multiple long hospital admissions. He had multiple chronic diseases, such as severe osteoporosis, fractures, renal stones, growth hormone deficiency, and electrolytes imbalance and anemia, all of which were complications of the nutritional deficiencies. Patient was kept inpatient and started on total parenteral nutrition (TPN) but this had an extreme effect on his mental health. He became depressed and had death ideations, then he refused to continue TPN. Two and a half years after his first presentation to our hospital, he presented to the ED with severe electrolyte disturbance and metabolic acidosis as well as abdominal pain, distension, and persistent vomiting. CT revealed small bowel obstruction with a transitional zone at the distal ileum. The patient underwent exploratory laparotomy, which showed a fibrous band at the terminal ileum that caused volvulus with significant proximal bowel dilatation. The band was released and the bowel appeared healthy. In addition, there was a suspicion of DCF between the right colon and the duodenum. The patient recovered smoothly. A small bowel study with gastrograffin showed a fistula between the first part of the duodenum and the hepatic flexure of the colon (Fig. 1A, B). The patient was counseled for definitive DCF repair after nutritional optimization.

**Fig. 2:**
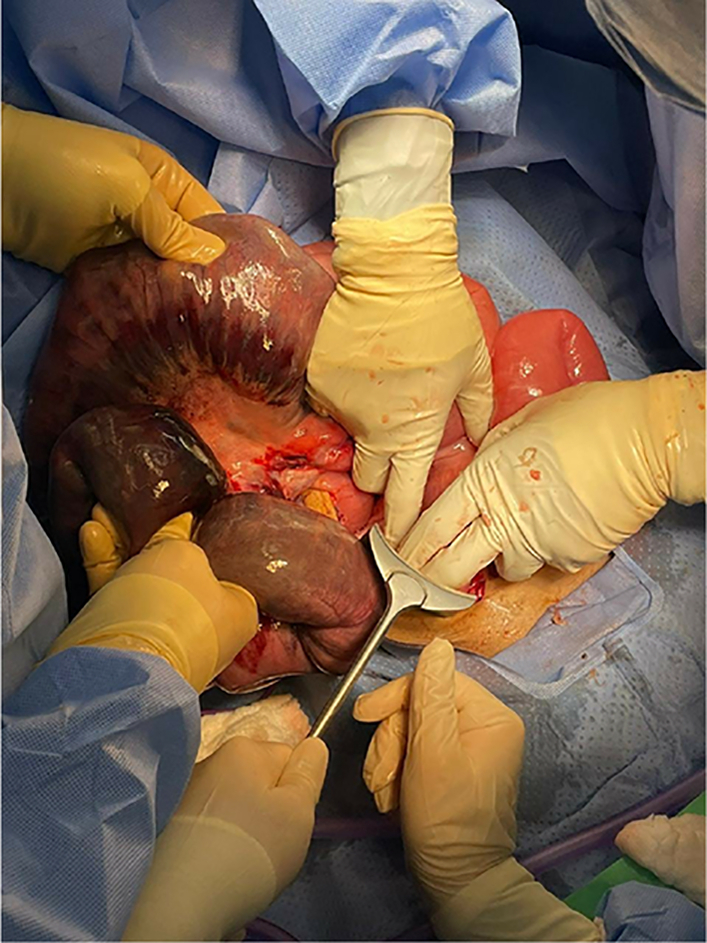
Twisted ischemic small bowel segment.

On the same admission, the patient underwent a trial of endoscopic nasojejunal tube insertion. Following the procedure and after 29 days of his first surgery, he experienced severe abdominal pain associated with tachycardia and hypotension. He was taken for exploratory laparotomy. A gush of foul-smelling fluid was noted upon abdominal entry. A long segment of twisted ischemic small bowel was identified and resected (Fig. 2).

**Fig. 3:**
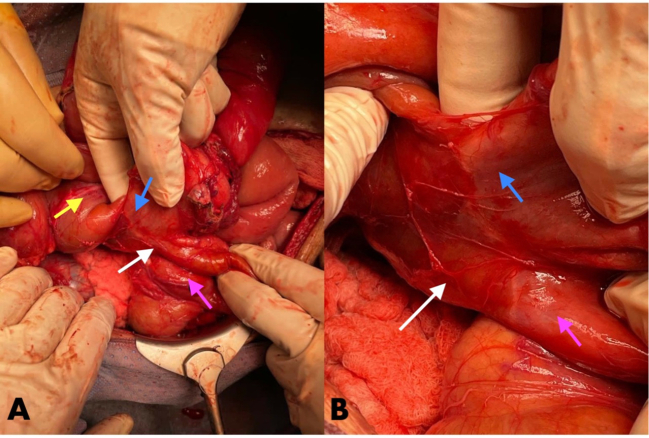
Intraoperative pictures showing the duodenocolic fistula. (A): A distant view of the duodenocolic fistula (white arrow); duodenum (pink arrow) and the colon (blue arrow) and the stomach (yellow arrow). (B): A closer view of the duodenocolic fistula (white arrow) can be visualized between the first part of the duodenum (pink arrow) and the hepatic flexure of the colon (blue arrow).

Right colon mobilization was done to explore the DCF. With meticulous dissection, the DCF was identified, a well-formed wide fistula tract with no active inflammation process. It was controlled with stapler and re-enforced second layer running suture (Fig. 3A, B).

Right hemicolectomy with side-to-side ileocolic anastomosis was then performed. The patient was extubated and shifted to the intensive care unit for five days, and then transferred to the general ward. Oral feeding was initiated immediately. The postoperative course was smooth, with no complications. The patient was discharged in a good condition after 14 days.

The patient was followed up regularly in the acute care surgery clinic. His last visit was nine months after surgery. All his chronic symptoms resolved. He had no more emergency department visits from the last surgery. His BMI increased from 11.5 to 20.6 kg/m^2^ over the nine months of follow-up.

## 3 Discussion

Long-standing DCF can have a major impact on a patient's health and quality of life. Early diagnosis and prompt surgical intervention can prevent many metabolic, nutritional, and psychological complications. In the setting of undiagnosed chronic gastrointestinal symptoms, DCF should be one of the differential diagnoses, especially if there is a history of bowel resection. In our case, we hypothesized that the cause of DCF was the bowel resection that the patient underwent in childhood since other causes of DCF had been ruled out.

Symptoms of DCF are non-specific and mimic those of other gastrointestinal disorders. The diagnosis is often delayed or missed [4]. Our patient did not experience fecal vomiting, which is one of the hallmarks of DCF. Fecal omitting happens as a result of high pressure in the large bowel that allows the retrograde flow of the large bowel contents back into the duodenum [3]. The absence of this symptom may be due to the fact that the pylorus can prevent the inverse passage of the duodenal contents into the stomach [4].

Our patient had a history of failure to thrive and malnutrition during infancy and childhood. DCF was unnoticed and misdiagnosed as functional gastrointestinal disorder (FGID). FGID, in terms of symptoms, presents like DCF [4], [8]. Okada et al. reported a case of a 14-year-old boy with chronic vomiting, diarrhea, and weight loss. The patient was misdiagnosed and treated as a case of FGID at a young age [4]. Then, he was managed as celiac disease during adulthood. Subsequently, DCF was discovered with colonic diverticulitis as the underlying etiology [4], [9].

Most of the gastrointestinal symptoms are explained by bacterial overgrowth in the small intestine, which eventually leads to malabsorption and malnutrition [3], [10]. Metabolic acidosis is a common consequence of DCF. It was first described by Benn et al. in 1997 as a result of the loss of alkaline pancreatic and biliary secretions into the lumen of the colon [11].

The preferred diagnostic method to detect DCF is barium enema. Sensitivity is as high as 90%, unlike barium meal and gastrografin swallow, which are reported to be less accurate [12]. Alternative methods that can be used to diagnose DCF are EGD, colonoscopy, CT, and magnetic resonance imaging [2]. Kamath et al., in 2011 concluded that a combination of endoscopy and radiologic imaging results in a complete evaluation of the position and etiology of the DCF [2]. However, multiple CT scans and endoscopy were performed in our case. None of them showed a fistula. It was detected using x-ray small bowel follow-through gastrografin study. Okada et al. reported false-negative results in the upper gastrointestinal contrast study. They noted the importance of the fistula location and the pressure gradient between the colon and duodenum. Therefore, they recommend the use of an effervescent agent with the upper gastrointestinal contrast to increase the pressure in the stomach and overcome the pressure in the colon. This allows good visualization of the fistula [4], [10].

The treatment of choice for DCF is surgery. It is also the only curative treatment. Surgeons face many challenges due to the complex anatomy around the duodenal region [13]. The selection of the surgical technique depends on the patient's condition and the underlying cause of the DCF. Colectomy with en bloc excision of the fistula is recommended [2]. The closure of the duodenal defect depends on its size. Small defects may be repaired with primary duodenal closure, while large defects require patch repair [2], [10], [14]. Roux-en-Y duodenojejunostomy and partial duodenal excision with gastrojejunostomy can be used as another method for large defects repair [2], [15].

In 1993, Michelassi et al. found that the rate of complications was approximately 33.3% following surgical treatment of DCF [15]. In our case, en bloc right hemicolectomy and excision of the fistula was performed with primary repair of the duodenal defect. We did not experience any complications during or after the surgery.

## 4 Conclusion

Patients with DCF are often very ill due to severe malnutrition, dehydration, and metabolic complications. Early diagnosis and intervention are the cornerstones of treatment. Resection of the diseased colon segment with fistula excision is the proper surgical intervention in most cases.

## Consent

The patients provided written informed consent for the publication of their clinical details and/or clinical photographs.

## Ethical approval

All procedures used in this study were approved by the ethics committee of our institution

## Funding

There was no funding body involved in the study's design, data collection, analysis, and interpretation, or manuscript writing.

## Guarantor

Ahmed A. Alburakan.

## Research registration number

1. Name of the registry: N/A
2. Unique identifying number or registration ID: N/A
3. Hyperlink to your specific registration (must be publicly accessible and will be checked): N/A.

## CRediT authorship contribution statement

Ahmed A. Alburakan: The corresponding author, operated the patients, revised the manuscript.
Sulaiman A. Alshammari: assessed the protocol, summed up, and wrote the manuscript, revised the manuscript, correction of the final paper.
Fayez G. Aldarsouni, MBBS: wrote the manuscript, followed up, revised the manuscript, correction of the final paper.
Thamer A. Nouh, MD: operated the patients, revised manuscript.

## Declaration of competing interest

The authors declare having no conflicts of interest for this article.
